# Prevention of cardiac tamponade by left intercostal Pericardiotomy for massive recurrent pericardial effusion: a case report

**DOI:** 10.1093/omcr/omae139

**Published:** 2024-11-25

**Authors:** Tomohiro Nakajima, Kei Mukawa, Hidemichi Kouzu, Ayaka Kamada, Nobuyoshi Kawaharada

**Affiliations:** Department of Cardiovascular Surgery, Sapporo Medical University School of Medicine, Sapporo, Japan; Department of Cardiovascular Surgery, Sapporo Medical University School of Medicine, Sapporo, Japan; Department of Cardiovascular, Renal and Metabolic Medicine, Sapporo Medical University School of Medicine, South-1, West-16, Chuo-ku, Sapporo 060-8543, Japan; Department of Cardiovascular, Renal and Metabolic Medicine, Sapporo Medical University School of Medicine, South-1, West-16, Chuo-ku, Sapporo 060-8543, Japan; Department of Cardiovascular Surgery, Sapporo Medical University School of Medicine, Sapporo, Japan

**Keywords:** cardiac tamponade, massive pericardial effusion

## Abstract

This case involved an 89-year-old woman with a history of left nephrectomy for left renal cell carcinoma at the age of 87 years. She had been gradually accumulating pericardial effusion for the past 4 years. She presented with signs of tachycardia and hypotension suggestive of cardiac tamponade due to pericardial effusion, and pericardiocentesis was performed below the xiphoid process in the cardiology department. Serous fluid was aspirated, and malignancy was ruled out by various tests. The patient subsequently developed recurrent pericardial effusion and was admitted to the hospital. Cardiovascular surgery was performed for pericardial drainage. A left intercostal incision was made for pericardiotomy and drainage of the pericardial effusion, allowing it to accumulate in the left pleural cavity in case of future accumulation. Pathological examination of the pericardium revealed no specific findings, and no cancer cells were present in the pericardial fluid. Prednisolone therapy was initiated for idiopathic pericarditis.

## Introduction

Cardiac tamponade is a condition characterized by the accumulation of a large amount of fluid within the pericardial sac. This leads to a highly urgent situation with a risk of shock due to decreased cardiac output from impaired diastolic filling and sudden cardiac arrest due to decreased coronary blood flow [1]. Under normal conditions, approximately 50 ml of pericardial fluid is present in the pericardial sac. However, acute cardiac tamponade can rapidly occur with even relatively small amounts of blood (approximately 100 ml) accumulating in the pericardial space secondary to conditions such as hemorrhage. Conversely, when pericardial fluid accumulates chronically over time, there may be no obvious clinical symptoms [2]. Causes of cardiac tamponade include acute pericarditis, malignant tumors, trauma, cardiac rupture following acute myocardial infarction, and acute aortic dissection, among others. The prevalence of idiopathic pericardial effusion is reported to be 2.6% of all pericardial effusions [3].

There are various methods for draining excessive pericardial effusion. If feasible, pericardial puncture drainage is performed via the subxiphoid approach [4]. If the subxiphoid approach is difficult, an alternative method involves making a small incision below the xiphoid process or performing a left thoracotomy under direct vision to access the pericardium and perform drainage [5]. Treatment decisions are made based on the patient’s condition and comprehensive assessment. In this case, we considered a surgical approach that takes into account the possibility of recurrent pericardial effusion leading to cardiac tamponade.

## Case report

**Figure 1:**
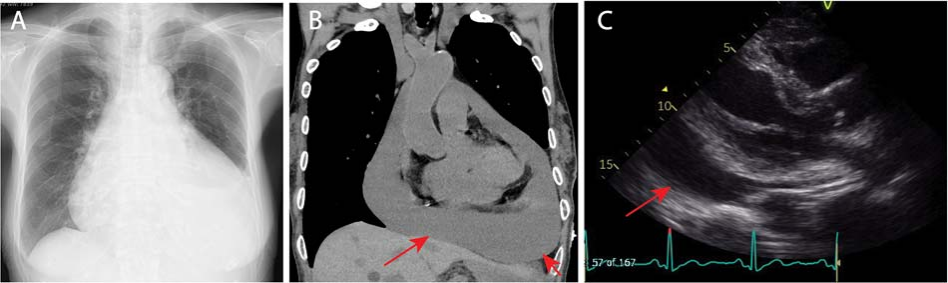
Preoperative images. (A) Preoperative chest X-ray. The cardiothoracic ratio was 0.70. (B) Preoperative computed tomography scan revealed massive cardiac effusion (arrow). (C) Preoperative echocardiogram revealed massive cardiac effusion (arrow).

**Figure 2:**
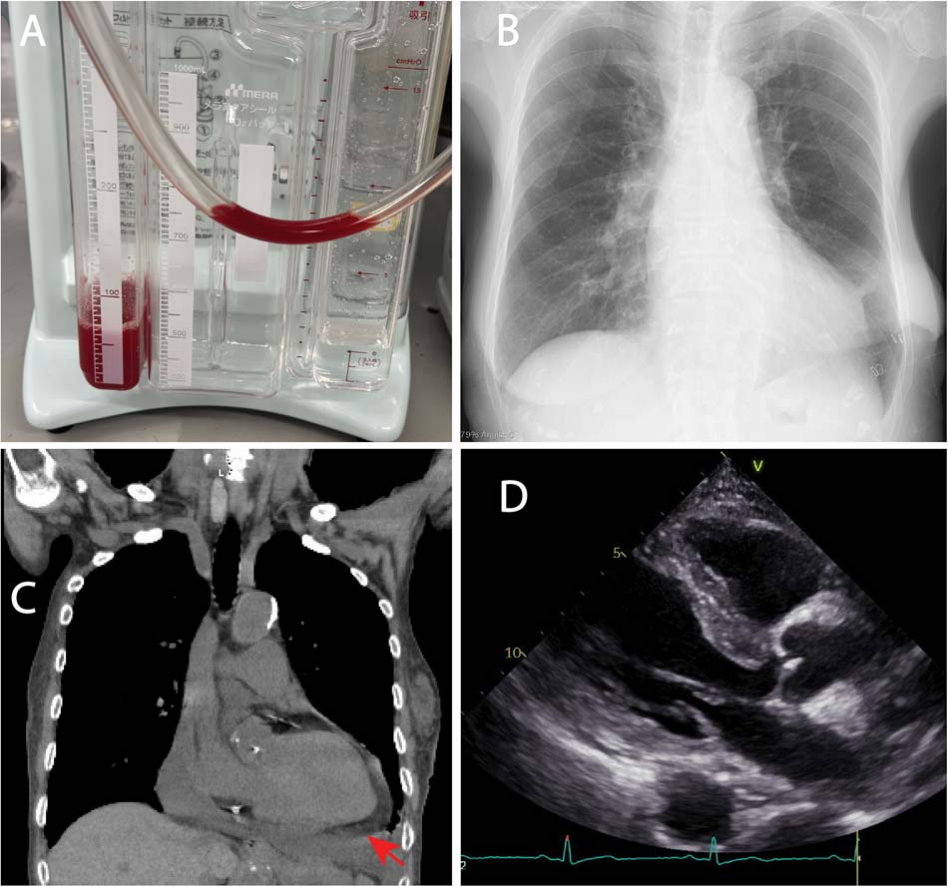
Postoperative images. (A) the pericardial effusion was slightly red. In total, 1200 ml of pericardial effusion was removed. (B) Postoperative chest X-ray. The cardiothoracic ratio was 0.45. (C) Postoperative computed tomography scan revealed little remaining cardiac effusion (arrow). (D) Postperative echocardiogram revealed little cardiac effusion.

This case involved an 89-year-old woman with a medical history of left carcinoma of the renal pelvis due to transitional epithelium at the age of 87 years, for which she underwent left nephrectomy, and atrial fibrillation treated with anticoagulant therapy. The patient had atrial fibrillation since the age of 85 years and was taking DOAC (edoxaban 15 mg). She had been experiencing a gradual accumulation of pericardial effusion for 4 years, which was being monitored in the outpatient setting. She developed recurrence of pericardial effusion that led to cardiac tamponade with symptoms of tachycardia and hypotension. Pericardiocentesis was performed below the xiphoid process in the cardiology department, yielding serosanguinous fluid. Cardiologists initiated colchicine 0.5 mg、Aspirin 900 mg. Subsequent investigations revealed no malignant findings. However, she was readmitted to the hospital 1 month later because of recurrent pericardial effusion. A chest X-ray revealed a cardiothoracic ratio of 0.70 and significant cardiac enlargement, and computed tomography showed approximately 40 mm of circumferential pericardial effusion (Fig. 1A-C). The patient had a blood pressure of 80/42 mmHg and heart rate of 110 beats/minute, indicating a pre-shock and cardiac tamponade state; therefore, she was referred to the cardiovascular surgery department for pericardial drainage. The surgical strategy involved a semi-urgent approach under general anesthesia. A 6-cm incision was made in the left fourth intercostal space, and upon reaching the left pleural cavity, the pericardium was opened. In total, 1200 ml of serosanguinous pericardial fluid was removed. Portions of the pericardium and pericardial fluid were submitted for pathological examination. Drains (19Fr Blake drain) were placed in the pericardial sac and left pleural cavity, and the surgery was completed. Pathological examination of the pericardium revealed no specific findings, and no cancer cells, no bacillus were detected in the pericardial fluid. The drains were removed on the fifth postoperative day. The patient was diagnosed with idiopathic pericarditis and started on prednisolone (20 mg/day) therapy. Subsequent follow-up showed no recurrence of pericardial effusion and no accumulation of left pleural fluid (Fig. 2A–D). After adjustment of her medication, the patient was discharged 28 days after surgery. Steroids are scheduled to be tapered off on an outpatient basis. Two weeks after discharge from the hospital, he visited the outpatient clinic for an echo and chest X-ray, which revealed no pericardial effusion. It has been two months since the surgery, and the steroid dose has been reduced to 10 mg.

## Discussion

According to Yamani et al., pericardiocentesis or indwelling catheter is the basic treatment for pericardial effusion in non-traumatic cases [5]. In the present case, pericardiocentesis was performed one month ago, and medical therapy for pericardial effusion was started, but pericardial effusion was observed again. Hemodynamically, the patient showed cardiac tamponade. Therefore, the first choice was a treatment plan that could be applied in the event of future pericardial effusions.

Cardiac tamponade is characterized by accumulation of fluid in the pericardial sac, which increases the pressure within the heart and prevents it from adequately expanding [1]. In other words, the heart is compressed by the surrounding fluid (pericardial fluid). As a result, the heart cannot function properly as a pump, leading to rapid shock, circulatory failure, and impaired consciousness. Cardiac tamponade is an emergency condition that can quickly lead to death. The usual surgical approach is to reach the pericardial sac by subxiphoid process or left open chest.

The causes of cardiac tamponade are varied. Cardiac tamponade may be idiopathic or caused by infection, acute aortic dissection, malignant tumors infiltrating the pericardium, collagen vascular diseases, drugs, trauma, and other conditions [6, 7]. In this case, despite various investigations, the final diagnosis was idiopathic pericarditis. Steroid therapy led to a reduction in the pericardial fluid. From a diagnostic and therapeutic perspective, the diagnosis of idiopathic pericarditis was appropriate [8].

Considering the patient’s medical history, she was at risk for recurrent pericardial effusion; thus, it was necessary to consider preventive measures. In this case, by approaching the heart from the left pleural cavity and excising the pericardium, we ensured that future accumulation of pericardial fluid could be managed as left pleural effusion. Treatment was initiated for idiopathic pericarditis, and the patient was discharged without recurrence of pericardial effusion or left pleural effusion. We considered treatment with steroids to decrease pericardial effusions, but in this particular case, the patient exhibited tachycardia and hypotension characteristic of cardiac tamponade, indicative of an impending shock state. The critical nature of the patient’s condition necessitated immediate intervention to relieve the tamponade. The hemodynamic instability precluded any consideration of steroid administration at that juncture. Our management priority was to promptly address the life-threatening cardiac tamponade to stabilize the patient’s condition.
